# Kidney biopsy findings in patients with obesity exhibit a wide spectrum of disease entities

**DOI:** 10.1093/ckj/sfag092

**Published:** 2026-03-19

**Authors:** Amélie Martinez, Pierre Letourneau, Maud Rabeyrin, Cécile Picard, Fitsum Guebre-Egziabher, Denis Fouque, Cyrielle Caussy, Christophe Soulage, Laetitia Koppe

**Affiliations:** Department of Nephrology, Hospices Civils de Lyon, Centre Hospitalier Lyon-Sud, Pierre-Bénite, France; Department of Nephrology, Hospices Civils de Lyon, Centre Hospitalier Lyon-Sud, Pierre-Bénite, France; Department of Pathology, Groupement Hospitalier Est, Hospices Civils de Lyon, Lyon, France; Department of Pathology, Groupement Hospitalier Est, Hospices Civils de Lyon, Lyon, France; CarMeN lab, INSERM U1060, INRA U1397, Université Claude Bernard Lyon 1, Pierrre-Bénite, France; Department of Nephrology, Hospices Civils de Lyon, Hôpital Edouard Herriot, Lyon, France; Department of Nephrology, Hospices Civils de Lyon, Centre Hospitalier Lyon-Sud, Pierre-Bénite, France; CarMeN lab, INSERM U1060, INRA U1397, Université Claude Bernard Lyon 1, Pierrre-Bénite, France; CarMeN lab, INSERM U1060, INRA U1397, Université Claude Bernard Lyon 1, Pierrre-Bénite, France; Department of Endocrinology, Hospices Civils de Lyon, Centre Hospitalier Lyon-Sud, Pierre-Bénite, France; CarMeN lab, INSERM U1060, INRA U1397, Université Claude Bernard Lyon 1, Pierrre-Bénite, France; Department of Nephrology, Hospices Civils de Lyon, Centre Hospitalier Lyon-Sud, Pierre-Bénite, France; CarMeN lab, INSERM U1060, INRA U1397, Université Claude Bernard Lyon 1, Pierrre-Bénite, France

**Keywords:** glomerulomegaly, kidney disease, obesity, obesity-related glomerulopathy, renal biopsy

## Abstract

**Background:**

Obesity is a growing public health concern and an independent contributor to chronic kidney disease, with obesity-related glomerulopathy (ORG) representing a distinct but insufficiently characterized entity. This study aimed to comprehensively describe the spectrum of kidney histological damage in patients with obesity and overt kidney disease.

**Methods:**

We reviewed 503 native kidney biopsies performed at Hospices Civils de Lyon between 2019 and 2022, identifying 103 adults with obesity. Glomerular histology was compared to 206 controls matched 2:1 for age, sex, hypertension, and diabetes.

**Results:**

In the cohort with obesity, mean body mass index (BMI) was 35.1 kg/m²; 83% had hypertension and 33% had diabetes. Biopsies were performed for active urinary sediment (56%), proteinuria (49%), and acute or chronic kidney failure (31% of cases each). The mean estimated glomerular filtration rate was 47 ml/min/1.73 m², and proteinuria was 3.13 g/day. The post-biopsy complication rate was similar between groups. A wide spectrum of renal lesions was observed: hypertensive nephrosclerosis (34%), acute tubular necrosis (34%), diabetic nephropathy (17%), IgA nephropathy (13%), and other glomerulonephritides. ORG was identified in 25.2% of cases, isolated in 6.8% and associated with other lesions in 18.4%, while 74.8% had alternative diagnoses. Although no significant difference in glomerular size was observed between patients with obesity and controls, glomerular diameter was positively correlated with BMI (*P* = .049).

**Conclusion:**

ORG is not the predominant cause of kidney disease in patients with obesity. Given the wide range of alternative diagnoses and the safety of the procedure, renal biopsy should be considered to guide accurate diagnosis and optimize management.

## GRAPHICAL ABSTRACT

**GRAPHICAL ABSTRACT:**
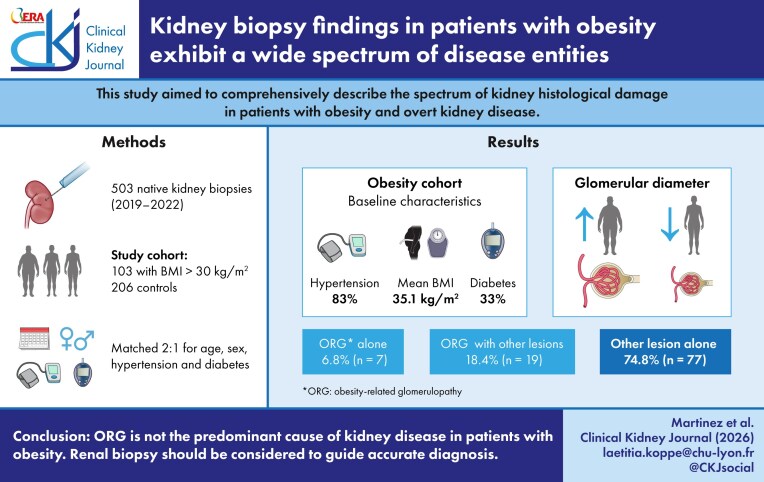

KEY LEARNING POINTS **What was known:**

- Obesity is a strong risk factor for chronic kidney disease (CKD), but the spectrum of histopathological lesions remains poorly defined.
- Obesity-related glomerulopathy (ORG) is considered a distinct entity, yet its prevalence and diagnostic criteria are debated.
- Most studies rely on US or Asian cohorts; European biopsy-based data are lacking.

**This study adds:**

- This is the first European renal biopsy-based study systematically characterizing kidney lesions in patients with obesity.
- ORG was uncommon (6.8% isolated), with alternative diagnoses such as hypertensive nephrosclerosis or diabetic nephropathy being more frequent.
- Glomerular size correlated with BMI, hypertension, and eGFR, suggesting subtle early structural changes in moderate obesity.

**Potential impact:**

- Nephrologists should not assume ORG as the main etiology in patients with obesity and kidney disease.
- Renal biopsy is feasible and safe in this population and can significantly alter management in one-third of cases.

## INTRODUCTION

Obesity represents a major global public health challenge, with its prevalence nearly tripling since 1975 worldwide [1]. In France, the 2020 ObEpi (Obesity Epidemiology)-Roche study reported that 47.5% of adults had a body mass index (BMI) exceeding 25 kg/m², and the prevalence of obesity has doubled since 1997, reaching 17%, with the most pronounced increase observed in class III obesity among younger populations [2].

Recently, a novel definition of obesity beyond the classical definition based solely on BMI has been proposed by a consensus of experts from the Lancet Commission. This novel definition reinforces the need to define clinical obesity as a chronic disease linked to the excess of adipose tissue and its impact on organ and tissue function, including chronic kidney disease (CKD) [3]. Numerous epidemiological studies have established a strong association between obesity and the development of CKD and/or albuminuria [4–9]. However, most of these investigations have relied exclusively on clinical and laboratory criteria for CKD, without addressing histological damage. Understanding the pathophysiological link between obesity and kidney injury remains complex due to frequent coexisting comorbidities such as hypertension and diabetes [10–14]. Nevertheless, several studies have shown an increased prevalence of kidney disease in individuals with obesity, even in the absence of hyperglycemia or hypertension [13, 14]. Moreover, obesity has been independently associated with the progression of IgA nephropathy, suggesting a direct pathogenic role [15, 16].

A growing body of evidence supports the concept of obesity as an independent risk factor for CKD progression and as a trigger for specific kidney lesions [17]. This hypothesis was first raised in 1974 by Weisinger *et al*., who described nephrotic syndrome in four patients with class III obesity [18]. Subsequent histological studies highlighted glomerulomegaly, with or without focal segmental glomerulosclerosis (FSGS), as a hallmark of what came to be known as obesity-related glomerulopathy (ORG) [19, 21]. The definition of glomerulomegaly is inconsistent across studies, depending on whether measurements are based on Bowman’s capsule or the floculus, and whether diameter, area, or volume is assessed. Proposed thresholds, derived from histologically normal kidney specimens [20], include a tuft diameter >180 µm, an area >30 000 µm², and a volume >3.27 × 10^6^ µm³, the latter estimate being calculated using the method developed by Wedeen and Gomez [22]. These values are commonly used as reference points in the literature, although no universally accepted standard has been established [14, 19, 20, 23, 24].

Data from kidney biopsies in individuals with class II or III obesity and without clinical evidence of kidney disease, particularly those undergoing bariatric surgery, remain scarce, especially within European cohorts [23, 24]. Recent analyses from two large American cohorts (*n* = 248, median BMI 44 kg/m²; and *n* = 255, mean BMI 40 kg/m²) identified ORG in only 30% and 40% of cases, respectively, highlighting a significant risk of underdiagnosis or diagnostic misclassification [25, 26]. To date, there is a lack of comprehensive cross-sectional, biopsy-based studies in Europe specifically focused on patients with obesity presenting with clinically overt kidney disease, and very few have incorporated matched of nonobese controls to allow comparative pathological evaluation.

To bridge this gap in knowledge, we conducted a cross-sectional study of kidney biopsy findings in patients with obesity undergoing clinically indicated biopsies over a 3-year period at Hospices Civils de Lyon, France. Our aim was to characterize the spectrum and relative frequency of kidney diseases, assess their clinical predictability, and report biopsy-related complications in this population.

## MATERIALS AND METHODS

### Patient selection, definition, and data collection

All native kidney biopsies performed on adult patients (age >18 years) and accessioned at the Renal Pathology Unit, Hospices Civils de Lyon (Lyon, France), between 1 May 2019, and 1 August 2022, were retrospectively reviewed to identify individuals with obesity, defined as a BMI >30 kg/m² (Supplementary Fig. S1). Patient charts were reviewed for body weight, height, and BMI (kg/m^2^) at the time of biopsy. Both height and weight were available for 489 patients. In accordance with the French Jardé law, this study was conducted within the framework of routine care and did not involve any additional procedures. Written information was provided to all subjects, and oral informed consent was required and obtained from all participants prior to inclusion. The study was approved by the institutional ethics committee of Hospices Civils de Lyon (Approval No. 22-5135).

The severity of obesity was stratified into three classes according to BMI: class I (30.0–34.9 kg/m²), class II (35.0–39.9 kg/m²), and class III (≥40.0 kg/m²).

Medical records were reviewed to extract demographic data (age, sex), as well as clinical history and comorbidities. Diabetes mellitus was defined by a fasting plasma glucose >1.26 g/l, and/or a random plasma glucose or an oral glucose tolerance test >2.0 g/l, and/or hemoglobin A1c (HbA1c) >6.5%, and/or the use of antidiabetic medications. Hypertension was defined as systolic blood pressure >130 mmHg and/or diastolic pressure >80 mmHg, or the use of antihypertensive drugs. Cardiovascular history included myocardial infarction, stroke, peripheral artery disease, angioplasty, and abdominal aortic aneurysm. Smoking status defined as current smoking or past smoking with cessation for more than 3 years, and alcohol use disorder was defined as >4 drinks/day or >14/week for men, and >3 drinks/day or >12/week for women. Dyslipidemia was defined by abnormal lipid profiles or the use of lipid-lowering therapy. History of obstructive sleep apnea syndrome (OSAS) was recorded if diagnosed by polysomnography or treated. Chronic corticosteroid use was defined as glucocorticoid therapy for more than 3 months.

We also recorded the presence of severe complications related to renal biopsy (e.g. need for blood transfusion, arterial embolization, or nephrectomy for hemostasis) and the presenting laboratory and serologic findings at the time of the procedure. The renal biopsy technique was also specified. All kidney biopsies were performed percutaneously, under either ultrasound guidance (by a nephrologist) or computed tomography guidance (by a radiologist) [27]. The choice of imaging guidance was determined by the nephrologist when kidney visualization by ultrasound was difficult or when anatomical particularities were present (e.g. multiple cysts). No transjugular or surgical renal biopsies were performed during this period.

We recorded the indications provided by the referring nephrologist for kidney biopsy: acute kidney injury (AKI) with previously normal renal function, AKI on a background of CKD, level of proteinuria, presence of a positive serologic test, and active urinary sediment. Definitions were based on Kidney Disease: Improving Global Outcomes (KDIGO) criteria [28]. Severe AKI (KDIGO stage 3) was defined as an increase of ≥26 µmol/l (0.3 mg/dl) of serum creatinine within 48 h, or a ≥50% rise in serum creatinine from baseline within 7 days, or a urine output ≤0.5 ml/kg/h for more than 6 h. Severe AKI (KDIGO stage 3) was defined as at least three-fold acute rise in serum creatinine from baseline, an acute serum creatinine level increase ≥354 µmol/l, or a urine output ≤0.3 ml/kg/h for more than 24 h, or anuria for ≥12 h. CKD was defined as an estimated glomerular filtration rate (eGFR) <60 ml/min/1.73 m², calculated using the Chronic Kidney Disease Epidemiology Collaboration equation, and/or the presence of markers of kidney damage for at least 3 months. Nephrotic-range proteinuria was defined as protein excretion ≥3.5 g/day or a urine protein-to-creatinine ratio ≥3.5 g/g. Nephrotic syndrome was defined as the combination of nephrotic-range proteinuria, serum albumin <30 g/l, and clinical edema. Active urinary sediment was defined as the presence of ≥10 red blood cells and/or ≥10 white blood cells per high-power field on microscopic examination. Urine protein electrophoresis abnormalities indicate the presence of Bence Jones proteinuria, which corresponds to a monoclonal component. A major therapeutic change was defined as the initiation, discontinuation, or modification of a specific treatment such as an immunosuppressive (e.g. corticosteroids, calcineurin inhibitors), an immunomodulatory (e.g. rituximab, mycophenolate mofetil), or an anti-inflammatory drug (e.g. colchicine, nonsteroidal anti-inflammatory drugs). Changes in nephroprotective therapies [e.g. renin–angiotensin–aldosterone system inhibitors (RAASi) or sodium–glucose co-transporter 2 inhibitors (SGLT2i)] were not considered major changes.

### Renal histopathology

All native kidney biopsies were processed using standard techniques for light microscopy (LM) and immunofluorescence. Histological interpretation was performed by one of four renal pathologists at the Renal Pathology Unit, Hospices Civils de Lyon (Lyon, France). With regard to the biopsies selected for this study, an independent pathological review was conducted by a second pathologist at the center to ensure diagnostic accuracy. Briefly, renal biopsies fixed in AFA, consisting of ethyl alcohol, formaldehyde, and acetic acid at an approximate volumetric ratio of 85:10:5, for 3–6 h, after which the tissue is routinely processed and embedded in paraffin for LM. Sectioned at 3–4 µm, and stained with hematoxylin and eosin, periodic acid–Schiff (PAS), Masson’s trichrome, and periodic acid–methenamine silver. Immunofluorescence labeling was performed on frozen kidney biopsy sections using antibodies against immunoglobulins (IgG, IgA, IgM), kappa and lambda light chains, fibrin, C3, and C1q. Noncontributive biopsies were defined as samples without glomeruli. Biopsy adequacy was assessed on LM sections according to Banff criteria: specimens were considered adequate if they contained ≥10 glomeruli and ≥2 arteries; minimal if they contained 7–9 glomeruli and 1 artery; and insufficient if 1–7 glomeruli and without an artery [29]. Global glomerulosclerosis was expressed as the percentage of globally sclerotic glomeruli relative to the total number of glomeruli. Interstitial fibrosis and tubular atrophy (IFTA) were scored semiquantitatively based on the percentage of affected cortical area: grade 0 (0%–5%), grade 1 (6%–25%), grade 2 (26%–50%), and grade 3 (>50%). If not explicitly scored, values were retrospectively estimated based on the descriptive pathology report. FSGS was classified according to the Columbia classification [tip, collapsing, cellular, perihilar, and not otherwise specified (NOS) variants].

Quantitative morphometric assessment was performed on one PAS-stained paraffin-embedded LM slide per patient using CaloPix Pocket software (version 4.1.0.9, TRIBVN Healthcare, Châtillon, France). For each glomerulus, the contours of the flocculus and Bowman’s capsule were manually traced to obtain the corresponding cross-sectional areas. From these measurements, the equivalent circular diameter was calculated using the formula $ECD = 2 \sqrt{A/\pi}$, where A is the measured area. To limit variability, all glomeruli present on the biopsy were analyzed per patient (mean of 7 in obesity cases and 6 in controls) and evaluated under blinded conditions by an independent renal pathologist. Globally sclerotic or tangentially sectioned glomeruli were excluded from the analysis.

Final kidney biopsy diagnoses, based on the main diagnostic line and pathologist comments, were categorized into three groups: (i) ORG alone; (ii) ORG associated with other kidney diseases; and (iii) other kidney diseases only. The pathological criteria for identifying ORG superimposed on another kidney lesion were established as the presence of pronounced and widespread glomerulomegaly, either isolated or accompanied by FSGS lesions disproportionate to the severity of the coexisting kidney disease. In the absence of formal pathological consensus on the definition of glomerulomegaly, we applied the historical cut-off of 180 µm for glomerular diameter, as proposed by Kambham *et al*. [20]. In our cohort, we used the same method and defined a study-specific threshold based on the mean glomerular tuft diameter plus one standard deviation, calculated from 37 selected lean individuals from the control cohort, all without diabetes, and/or hypertension. The diagnosis of diabetic nephropathy was established according to the 2010 Renal Pathology Society classification. Hypertensive nephrosclerosis was diagnosed in patients with a documented history of longstanding hypertension together with characteristic histopathological features, including moderate-to-severe arteriosclerosis and arteriolosclerosis, diffuse global glomerulosclerosis, ischemic glomerular tuft retraction, and subcapsular fibrosis. In cases with ambiguous clinico-pathological correlation and overlapping features of ORG, an independent pathological review was performed to refine the diagnosis.

### Statistical analyses

Statistical analyses were performed using R statistical software (version 4.2.1). Values are expressed as mean ± range [min-max] or standard deviation [SD], unless otherwise specified. Matching between obesity cases and controls without obesity in a 1:2 ratio was performed using propensity score matching based on sex, age, and the presence of hypertension and diabetes. Matching was conducted using the nearest neighbor algorithm implemented in the MatchIt package of R. Comparisons between two groups (obesity vs. controls) were conducted using the Wilcoxon rank-sum test for continuous variables and the chi-squared test for categorical variables. For comparisons across more than two groups (e.g. obesity classes), analysis of variance was used for continuous variables, and the chi-squared test for categorical variables. Several linear regression models were constructed to assess the association between BMI and glomerular size. Variables known from the literature to potentially influence glomerular morphology were first included in univariate analyses and in an initial multivariable model. A second multivariable model was then constructed using stepwise selection (both forward and backward) to identify the most parsimonious set of predictors. A two-sided *P*-value <.05 was considered statistically significant.

## RESULTS

### Cohort characteristics

Between 1st May 2019, and 1st August 2022, 503 native kidney biopsies were processed at the Renal Pathology Unit, Hospices Civils de Lyon. After excluding 7 patients with multiple biopsies, 17 with nonrepresentative samples [2.5% (*n* = 9) without obesity, 7.2% (*n* = 8) with obesity], 14 with missing BMI data, and 10 kidney transplant recipients, 455 patients were retained for analysis. Among them, 103 (22%) had a BMI >30 kg/m². Patients with obesity were matched 1:2 to 206 controls without obesity based on age, sex, and the presence of hypertension and diabetes (Supplementary Fig. S1 and Table 1). Compared to patients with obesity, control patients had significantly lower rates of dyslipidemia and OSAS, and more frequent hematuria, glucagon-like peptide-1 receptor (GLP1) agonists treatment and abnormal serologic findings.

The matched control cohort (*n* = 206) had a mean age of 53 years [range: 18–89] and a mean BMI of 24 kg/m² [range: 16–30] (Table 1). The cohort of patients with obesity (*n* = 103) had a mean age of 52 years [range: 19–84] and a mean BMI of 35.1 kg/m² [30.0–56.6]. Clinical characteristics were broadly similar across obesity classes, except for a higher proportion of women in classes II and III. Comorbidities commonly associated with obesity were prevalent: hypertension (83%), dyslipidemia (59%), diabetes mellitus (35%, mean duration 13 years), and OSAS (22%). Diabetic retinopathy was present in 8.7% of diabetic patients; however, ophthalmologic assessments were available for only 66% of the cohort. Three patients had undergone bariatric surgery prior to biopsy. At the time of biopsy, 59% were receiving RAASi, 2% were receiving SGLT2i and 7.8% were receiving GLP1 agonist. Renal function was impaired in most cases, with a mean eGFR of 47 ml/min/1.73 m² [3–90]. Nephrotic-range proteinuria was observed in 38% of patients (mean proteinuria: 3.13 g/day or g/g; [0–17.2]), with a mean serum albumin of 33 g/l [13–45]. Hematuria was present in 53% of patients. Abnormal immunologic tests were observed in 43% of cases, most commonly positive antinuclear antibodies (19%) and abnormal serum protein electrophoresis or free light chain ratios (19%) (Table 1).

**Table 1:** Demographic and clinical characteristics of the 103 patients with obesity and 206 matched control patients undergoing renal biopsy, stratified by obesity class.

	Obesity class							
Characteristics	Class I (*N* = 65)	Class II (*N* = 20)	Class III (*N* = 18)	Overall (*N* = 103)	Matched control patients (*N* = 206)	*P*-value^a^	*P*-value^b^	% missing value
Age, years	50 [19–84]	54 [27–77]	56 [24–82]	52 [19–84]	53 [18–89]	.5	.5	0
Female	25 (38)	12 (60)	13 (72)	50 (49)	97 (47)	.021	.80	0
BMI, kg/m^2^	32 [30–34.6]	36.8 [35–39.1]	44.8 [40–57]	35.1 [30–56.6]	24 [16–30]	<.001	<.001	0
Diabetes	21 (32)	7 (35)	8 (44)	36 (35)	70 (34)	.6	.8	0
Diabetes history, years	12 [1–44]	12 [12–26]	18 [6–33]	13 [1–44]	12 [0–59]	.3	.4	5
Diabetic retinopathy	3 (4.6)	5 (25)	1 (5.6)	9 (8.7)	20 (9.7)	.006	<.001	33
Hypertension	53 (82)	17 (85)	15 (83)	85 (83)	167 (81)	>.9	>.9	0
Cardiovascular events	7 (11)	1 (5)	0 (0)	8 (7.8)	29 (14)	.5	.3	1
Active smoking	13 (20)	4 (20)	4 (22)	21 (21)	44 (21)	>.9	>.9	1
Alcoholism (past or active)	3 (4.7)	4 (20)	1 (5.6)	8 (7.8)	18 (8.7)	.085	.4	0
Dyslipidemia	34 (52)	15 (75)	10 (56)	59 (57)	67 (33)	.2	<.001	0
OSAS	14 (22)	4 (20)	5 (28)	23 (22)	11 (5.3)	.8	<.001	0
MASLD	4 (6.2)	3 (15)	3 (17)	10 (9.7)	10 (4.9)	0.2	.066	0
After bariatric surgery	3 (4.6)	0 (0)	0 (0)	3 (2.9)	0 (0)	>.9	>.9	0
RAASi treatment	37 (57)	13 (65)	11 (61)	61 (59)	128 (62)	.8	.9	0
SGLT2i treatment	1 (1.5)	0 (0)	1 (5.5)	2 (1.9)	2 (1)	.4	.6	0
GLP1 agonist treatment	5 (7.7)	1 (5)	2 (11)	8 (7.8)	4 (1.9)	.7	.02	0
Glucocorticoids treatment	11 (17)	2 (10)	1 (5.6)	14 (14)	43 (21)	.6	.3	0
Serum creatinine, µmol/l	194 [35–729]	173 [38–492]	238 [35–1050]	198 [35–1050]	185 [32–1690]	.7	.8	0
eGFR, ml/min per 1.73 m^2^	47 [5–90]	49 [10–90]	44 [3–90]	47 [3–90]	41 [5–103]	.8	.4	0
Proteinuria, g/d or g/g creatinine	2.9 [0–17.2]	4 [0–14.7]	2.9 [0.2–12.8]	3.1 [0–17.2]	2.77 [0–15.80]	.5	.6	1
Hematuria	33 (51)	13 (65)	9 (50)	55 (53)	140 (68)	.2	.045	2
Serum albumin, g/l	34 [13–45]	32 [17–45]	32 [21–42]	33 [13–45]	32 [0–54]	.14	.2	5
HbA1c, %	6.9 [0–12.8]	6.8 [5.2–11.5]	6.4 [4.9–9]	6.8 [0–12.8]	6.09 [0–11.90]	.8	.3	51
Uricemia, µmol/l	466 [0–844]	408 [119–651]	436 [214–648]	449 [0–844]	407 [0–725]	.3	.027	28
Abnormal serology result				44 (43)	169 (83)		<.001	
(+) ANA or dsDNA	13 (20)	4 (20)	3 (17)	20 (19)	48 (23)	>.9	<.001	15
(+) ANCA or anti-GMB	7 (11)	2 (10)	1 (5.6)	10 (9.8)	21 (10)	>.9	<.001	24
SPEP or FLC ratio abnormality	10 (16)	4 (20)	5 (28)	19 (19)	26 (13)	.7	.005	10
UPEP abnormality	4 (6.6)	1 (5)	0 (0)	5 (5.1)	7 (3.4)	.6	<.001	46
Anti-PLA2R	0 (0)	1 (5)	0 (0)	1 (1)	2 (1.0)	.4	<.001	53
Cryoglobulinemia	5 (7.7)	2 (10)	2 (12)	9 (8.8)	27 (13)	>.9	<.001	33
Low complements	8 (12)	1 (5)	1 (5.6)	10 (9.7)	38 (18)	.8	<.001	17

Values presented are mean [range] or N (%).

a *P* values reflect comparisons between the three obesity groups (class I, class II, and class III), using analysis of variance (ANOVA) for continuous variables and the Chi-squared test for categorical variables.

b *P* values reflect comparisons between patients with obesity and matched controls without obesity using the Wilcoxon rank-sum test for continuous variables and the Chi-squared test for categorical variables.

ANA: antinuclear antibody; ANCA: antineutrophil cytoplasmic antibody; Anti-PLA2R: anti-phospholipase A2 receptors antibodies; BMI: body mass index; dsDNA: double-stranded DNA; eGFR: estimated glomerular filtration rate according to Chronic Kidney Disease Epidemiology Collaboration (CKD-EPI) equation; FLC: free light chain; HbA1c: hemoglobin A1C; GLP1: glucagon-like peptide-1; MASLD: meta bolic dysfunction–associated steatotic liver disease; OSAS: obstructive sleep apnea syndrome; SGLT2i: sodium-glucose co-transporter 2 inhibitors; RAASi: renin-angiotensin-aldosterone system inhibitor; SPEP: serum protein electrophoresis; UPEP: urine protein electrophoresis.

### Kidney biopsy indications and complications in the cohort with obesity

In patients with obesity, kidney biopsy was performed for one or several clinical indications, most commonly active urinary sediment (56%), proteinuria (49%), AKI (31%), and CKD (31%) (Table 2). Given the technical constraints in this population, kidney biopsies were more frequently performed under computed tomography guidance by radiologists in patients with more severe obesity compared with the control group (*P* < .001) (Table 3). Complication rates were low (7%) and exclusively minor, including six cases of perirenal hematoma and one case of macroscopic hematuria. Importantly, when compared to matched controls, no significant differences were observed in complication rates (Table 3). According to the Banff criteria, biopsy sample adequacy was similar between groups, with 22% of specimens deemed insufficient in patients with obesity and 18% in controls (*P* = .48) (Table 3). However, we observed a significantly higher number of noncontributive biopsies in patients with obesity (*P* = .02, Supplementary Fig. S1). The adequacy rate of biopsies did not differ when the procedure was performed by a radiologist using computed tomography guidance (five of nine biopsies were adequate in patients with obesity vs. five of six in the control group, *P* = .26). Kidney biopsy led to a major therapeutic modification in 35% of patients with obesity, beyond standard conservative measures such as RAASi, SGLT2i, statins, or diuretics already in place for CKD management (Table 2).

**Table 2:** Biopsy indications and complications in 103 patients with obesity.

	Obesity class				
	Class I (*N* = 65)	Class II (*N* = 20)	Class III (*N* = 18)	Overall (*N* = 103)	*P*-value^a^
Active urine sediment	35 (54)	15 (75)	8 (44%)	58 (56)	.3
Proteinuria^b^	33 (51)	8 (40)	9 (50)	50 (49)	.7
AKI	19 (29)	6 (30)	7 (39)	32 (31)	.7
CKD	23 (35)	6 (30)	3 (17)	32 (31)	.3
AKI on CKD	10 (15)	3 (15)	3 (17)	16 (16)	>.9
Nephrotic range proteinuria without nephrotic syndrome	9 (14)	5 (25)	5 (28)	19 (18)	.3
Nephrotic syndrome	12 (18)	6 (30)	2 (11)	20 (19)	.4
Other reason for biopsy	4 (6.2)	2 (10)	1 (5.6)	7 (6.8)	>.9
Major therapeutic change	26 (40)	5 (25)	5 (28)	36 (35)	.4

Values presented are *N* (%).

a *P* values reflect comparisons between the three obesity groups (class I, class II, and class III), using the Chi-squared test for categorical variables.

b Proteinuria: **<**3.5 g/day or a urine protein-to-creatinine ratio (UPCR) <3.5 g/g.

AKI: acute kidney injury; CKD: chronic kidney disease according to Kidney Disease Improving Global Outcome definition.

**Table 3:** Biopsy complications and adequacy in 103 patients with obesity and 206 matched controls.

	Obesity class						
	Class I (*N* = 65)	Class II (*N* = 20)	Class III (*N* = 18)	Overall (*N* = 103)	Controls (*N* = 206)	*P*-value^a^	*P*-value^b^
Radiologist guided biopsy	2 (3.1)	0 (0)	7 (39)	9 (8.7)	6 (2.9)	<.001	.024
Post biopsy complications						.5	
None	61 (94)	18 (90)	17 (94)	96 (93)	188 (91.3)		.55
Peri-renal hematoma	4 (6.2)	1 (5)	1 (5.6)	6 (5.8)	6 (2.9)		.212
Macroscopic hematuria	0 (0)	1 (5)	0 (0)	1 (1)	12 (5.8)		.04
Inadequate kidney biopsy samples	14 (22)	4 (20)	5 (28)	23 (22)	39 (18.9)	.13	.48

Values presented are mean [standard deviation] or N (%).

a *P* values reflect comparisons between the three obesity groups (class I, class II, and class III), using analysis of variance (ANOVA) for continuous variables and the Chi-squared test for categorical variables.

b *P* values reflect comparisons between patients with obesity and matched controls without obesity , using the Wilcoxon rank-sum test for continuous variables and the Chi-squared test for categorical variables.

### Impact of obesity on histopathological findings

Six of the 309 biopsies were excluded due to insufficient glomerular tissue (all in the control group). Among the 103 patients with obesity, histological analysis showed heterogeneous IFTA severity, predominantly grades II and III, with 26% global glomerulosclerosis and FSGS in 57% of cases, mainly of the NOS subtype (Table 4). Compared to matched controls, glomerular dimensions did not differ significantly (Table 5).

**Table 4:** Histopathological findings in 103 patients with obesity.

	Obesity class				
	Class I (*N* = 65) [1]	Class II (*N* = 20) [1]	Class III (*N* = 18) [1]	Overall (*N* = 103) [1]	*P*-value [2]
IFTA, %					.5
0 (0%–5%)	5 (7.7)	1 (5)	2 (11)	8 (7.8)	
1 (6%–25%)	22 (34)	10 (50)	6 (33)	38 (37)	
2 (26%–50%)	19 (29)	3 (15)	2 (11)	24 (23)	
3 (>50%)	19 (29)	6 (30)	8 (44)	33 (32)	
Global glomerulosclerosis, %	24 [21]	23 [16]	33 [28]	26 [22]	.6
FSGS	38 (58)	11 (55)	10 (56)	59 (57)	>.9
NOS	32 (49)	9 (45)	9 (50)	50 (49)	>.9
Peri-hilar	4 (6.2)	0 (0)	2 (11)	6 (5.8)	.3
Tip-variant	2 (3.1)	2 (10)	1 (5.6)	5 (4.9)	.3
Number of glomerulus	7.4 [4.7]	6.4[4.1]	4.4 [3.1]	6.7 [4.5]	.04
Mean floculus diameter, µm	160 [27]	168 [22]	170 [34]	163 [27]	.2
Mean Bowman’s capsule diameter, µm	189 [27]	196 [23]	200 [34]	192 [28]	.2
Mean floculus area, µm^2^	20.820 [7.817]	22.661 [5.854]	23.302 [9.501]	21.611 [7.803]	.14
Mean Bowman’s capsule area, µm^2^	28.748 [9.043]	31.088 [6.873]	32.154 [11.706]	29.798 [9.217]	.11

Values presented are mean [standard deviation] or *N* (%).

a *P* values reflect comparisons between the three obesity groups (class I, class II, and class III), using analysis of variance (ANOVA) for continuous variables and the Chi-squared test for categorical variables.

IFTA: interstitial fibrosis tubular atrophy; FSGS: focal segmental glomerulosclerosis.

**Table 5:** Glomerular dimensions in 103 patients with obesity and 206 matched control patients.

	Cases (*N* = 103)	Controls (*N* = 200)^a^	*P*-value^b^
Number of glomeruli	6.7 [4.5]	6.2 [4.3]	.19
Mean floculus area, µm^2^	21.611 [7.803]	21.743 [8.453]	.9
Mean Bowman’s capsule area, µm^2^	29.798 [9.217]	32.691 [52.438]	.4
Mean floculus diameter, µm	163 [27]	161 [26]	.6
Mean Bowman’s capsule diameter, µm	192 [28]	190 [28]	.5

Values presented are mean [standard deviation].

a Six biopsies were excluded due to insufficient glomerular tissue for analysis.

b *P* values reflect comparisons between patients with obesity and matched controls without obesity, using the Wilcoxon rank-sum test.

A positive association between BMI and glomerulomegaly (based on Bowman’s capsule area) was found (Table 6 and Supplementary Fig. S2). After multivariable adjustment, glomerulomegaly was independently associated with continuous variables such as BMI (*P* = .049; β = 0.005) and eGFR (*P* = .030; β = 0.001), as well as with hypertension (*P* = .008; β = 0.090) (Table 3). In 37 lean controls without diabetes or hypertension, the mean flocculus diameter was 150.2 ± 17.3 µm. These findings suggest that moderate obesity may be linked to early and subtle glomerular structural changes, and justify a refined glomerulomegaly threshold of 168 µm.

**Table 6:** Predictive factors of glomerulomegaly using multivariate logistic regression.

	Univariate model			Multivariates model full			Multivariates model step		
Variables	Beta	95% CI	*P*-value	Beta	95% CI^1^	*P*-value	Beta	95% CI	*P*-value
BMI	0.005	0.000, 0.010	**.042**	0.005	0.000, 0.010	**.033**	0.005	0.000, 0.009	**.049**
Hypertension	0.098	0.033, 0.163	**.004**	0.085	0.016, 0.154	**.016**	0.090	0.024, 0.156	**.008**
Diabetes	0.054	0.001, 0.107	**.048**	0.054	−0.004, 0.113	.067	0.047	−0.007, 0.102	.088
eGFR	0.001	0.000, 0.002	.14	0.001	0.000, 0.002	.051	0.001	0.000, 0.002	**.030**
OSAS	−0.007	−0.007, 0.055	.8	−0.043	−0.108, 0.022	.2	−0.041	−0.101, 0.019	.2
Age	0.000	−0.001, 0.002	>.9	0.000	−0.002, 0.001	.6			
Male sex	−0.013	−0.065, 0.039	.6	−0.024	−0.077, 0.028	.4			

Predictive factors of log (mean Bowman’s capsule area) in a sample of 103 obese patients, Coefficients (β), 95% confidence intervals (CI), and *P*-values are presented for univariate, full multivariate, and stepwise AIC-selected multivariate linear regression models. BMI: body mass index; eGFR: estimated glomerular filtration rate; OSAS: obstructive sleep apnea syndrome.

### Spectrum of kidney disease in patients with moderate obesity

Kidney biopsies in patients with obesity revealed a broad range of histopathological diagnoses with a diversity similar to that observed in matched control patients. Nonmutually exclusive etiologies included hypertensive nephrosclerosis (34%), acute tubular necrosis (34%), diabetic nephropathy (17%), IgA nephropathy (13%), interstitial nephritis (9.7%), and lupus nephritis (9.7%), with no significant variation across obesity classes. Although 43% of patients had serological abnormalities, only 18% had biopsy-proven immune-mediated kidney disease, predominantly lupus nephritis or monoclonal gammopathy of renal significance (Supplementary Table S1).

A pattern consistent with ORG was identified in 25% and 39% of cases using glomerular diameter cut-offs of 180 µm and 168 µm, respectively, with no variation across obesity class (Supplementary Table S1). Using the 180 µm threshold, ORG was more frequently observed in combination with another renal lesion (*n* = 19; 18.4%) than as an isolated finding (*n* = 7; 6.8%), while 77 patients (74.8%) showed no ORG features. Compared to patients with other lesions, those with isolated ORG exhibited higher eGFR, lower prevalence of dyslipidemia, and more frequent use of RAASi. Diabetes prevalence, use of SGLT2i, and use of GLP-1 agonist did not differ between groups (Table 7).

**Table 7:** Repartition between obesity-related glomerulopathy (ORG) and differential diagnosis.

Characteristics	ORG alone (*N* = 7)	ORG with other lesion (*N* = 19)	Other lesion alone (*N* = 77)	*P*-value^a^
Age, years	40.6 [14.7]	50.9 [16.0]	53.6 [17.5]	.1513
Female	3 (42.9)	11 (57.9)	36 (46.8)	.6523
BMI, kg/m^2^	38.2 [9.12]	36.2 [5.8]	34.6 [4.7]	.3417
Diabetes	2 (28.6)	10 (52.6)	24 (31.2)	.1997
Hypertension	5 (71.4)	18 (97.7)	62 (80.5)	.2494
Cardiovascular events	0 (0)	0 (0)	8 (10.4)	.2312
Active smoking	1 (14.3)	5 (26.3)	22 ()	.715
Alcoholism (past or active)	0 (0)	0 (0)	7 (9.2)	.2814
Dyslipidemia	2 (28.6)	15 (78.9)	42 (54.6)	**.0442**
OSAS	1 (14.3)	4 (21.1)	18 (23.4)	.8489
MASLD	0 (0)	2 (10.5)	8 (10.4)	.6677
After bariatric surgery	0 (0)	0 (0)	3 (3.9)	.5935
RAASi treatment	2 (28.6)	16 (91.7)	43 (55.8)	**.0183**
SGLT2i treatment	0 (0)	1 (5.2)	1 (1.3)	.440
GLP1 agonist treatment	0 (0)	2 (10.5)	6 (7.8)	.801
Glucocorticoids treatment	1 (14.3)	3 (15.8)	10 (13)	.9489
eGFR, ml/min per 1.73 m^2^ via CKD-EPI	74.9 [26.2]	52.2 [27.6]	43.3 [27.4]	**.0203**
Proteinuria, g/d or g/g creatinine	2.3 [2.3]	3.3 [3.3]	3.1 [3.7]	.5290
Hematuria	4 (57.1)	8 (42.1)	43 (56.6)	.5493
Serum albumin, g/l	38.4 [4.4]	33.8[8.6]	32.8 [8.3]	.2386
HbA1c, %	5.8 [0.5]	7.5 [2.1]	6.6 [2.3]	.1363
Uricemia, µmol/l	386 [145]	411 [122]	466 [147]	.2098
Abnormal serology result	3 (42.9)	10 (52.6)	30 (39)	.5557

Values presented are mean [standard deviation] or *N* (%).

a *P* values reflect comparisons between the three groups (ORG alone, ORG with other lesion, and other lesion alone), using analysis of variance (ANOVA) for continuous variables and the Chi-squared test for categorical variables.

BMI: body mass index; eGFR: estimated glomerular filtration rate according to Chronic Kidney Disease Epidemiology Collaboration (CKD-EPI) equation; HbA1c: hemoglobin A1C; GLP1: glucagon-like peptide-1; iSGLT2i: sodium-glucose co-transporter 2 inhibitors; MASLD: Metabolic dysfunction–Associated Steatotic Liver Disease; ORG: obesity-related glomerulopathy; OSAS: obstructive sleep apnea syndrome; RAASi: renin-angiotensin-aldosterone system inhibitor.

**Figure 1:**
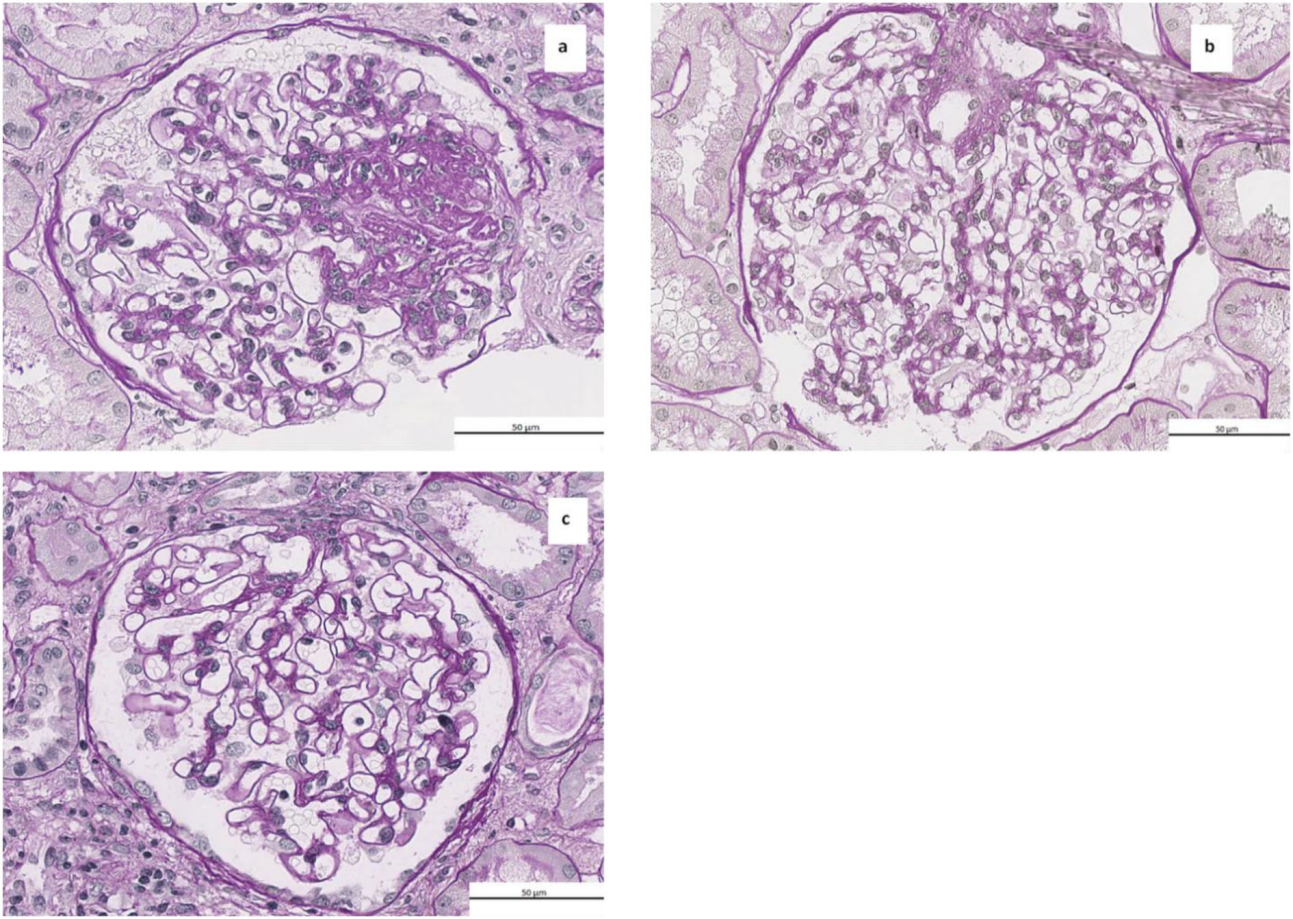
Biopsy findings among patients with obesity related glomerulopathy compared to a lean control patient without glomerulomegaly. (a) ORG presenting with glomerulomegaly and focal segmental glomerulosclerosis (FSGS) not otherwise specified (NOS) (floculus diameter of 215 µm) (periodic acid-Schiff (PAS), original magnification × 400). (b) Obesity related glomerulopathy (ORG) showing typical glomerulomegaly without FSGS (floculus diameter of 214 µm) (PAS, original magnification × 400). (c) Absence of glomerulomegaly (floculus diameter of 153 µm) (PAS, original magnification × 400).

Among patients with isolated ORG, glomerulomegaly with FSGS was the predominant lesion (Fig. 1 and Supplementary Table S2). The most common co-existing lesions with ORG were hypertensive nephrosclerosis (*n* = 10) and diabetic nephropathy (*n* = 7). In contrast, the majority of biopsies without ORG features (*n* = 77) revealed hypertensive nephrosclerosis (*n* = 29), diabetic nephropathy (*n* = 10), or IgA nephropathy (*n* = 11) (Supplementary Table S2).

## DISCUSSION

This study provides the first histopathological characterization of kidney disease in a French cohort of patients with mild to moderate obesity, based on reproducible morphometric data.

In 2020, class III obesity represented only 2% of the European population, compared to 9.2% in the USA in 2017–2018 (National Center for Health Statistics) [30, 31]. Ethnic differences in metabolic risk, such as a higher susceptibility in Asian populations at equivalent adiposity, further support the need for European-specific data [19, 32]. ORG remains underdiagnosed, despite a reported 10%–33% risk of progression to kidney failure requiring kidney replacement therapy at 6 years in some cohorts [21, 33]. In our cohort, only 6.8% of patients with obesity had isolated ORG, with no clear distinguishing clinical or biological features. Biopsy impacted the management of kidney disease in 35% of cases. Despite technical challenges, complication rates did not exceed those of standard practice [34] and sample quality was similar to that of the control group. However, maintaining biopsy quality may be related to the higher number of procedures performed under computed tomography guidance. These findings underscore the relevance and safety of kidney biopsy for guiding care in patients with obesity. They also suggest that computed tomography guidance should be considered in cases of technical difficulty or insufficient visualization to ensure successful completion of the procedure.

Our findings are consistent with a recent retrospective study by Choung *et al*., involving 248 American patients with class II and III obesity (mean BMI 44 kg/m²) who underwent clinically indicated kidney biopsy [26]. ORG was present in one-third of cases—isolated in 12% and associated with other lesions in 18.4%—and accounted for 69% of differential diagnoses, often diabetic nephropathy or hypertensive nephrosclerosis. In half of the cases, the diagnosis led to therapeutic modifications. However, glomerulomegaly assessment was based solely on qualitative morphology, introducing pathologist-dependent variability. An eGFR <30 ml/min/1.73 m² and nephrotic syndrome were associated with a higher likelihood of non-ORG diagnoses. Acute tubular necrosis was frequent (15%, *n* = 35), suggesting increased tubulo-interstitial vulnerability in this population. Similarly, Salvatore *et al*. reviewed 255 native biopsies in patients with severe obesity (mean BMI 40.3 kg/m²) and found a diagnosis other than ORG in 60% of cases [25]. No significant differences in glomerular size or FSGS prevalence were observed between subgroups of obesity.

The clinical presentation of isolated ORG in our study aligns closely with the classical description: mild proteinuria, preserved serum albumin, and mild to moderate decline in GFR [20]. The hyperfiltration typically associated with obesity is reflected in higher eGFR values in patients with isolated ORG. In contrast, proteinuria ≥3 g/day and eGFR <45 ml/min/1.73 m² were more frequently observed in cases with alternative or additional diagnoses. This must be interpreted cautiously, as creatinine-based equations indexed to body surface area may underestimate GFR in individuals with obesity, a limitation well-documented in the literature [35–37]. Nonetheless, our limited cohort, with few cases of isolated ORG, precluded the identification of subtle clinical features specific to ORG or predictive markers for differential diagnosis, reinforcing the need for histopathological confirmation.

In obesity, renal injury and glomerular changes are driven by insulin resistance, chronic inflammation, RAAS hyperactivation with hypertension, glomerular hyperfiltration, and nonesterified free fatty acids-induced lipotoxicity [38–40]. In our study, glomerular morphometric measurements did not significantly differ between cases and controls, despite matching for potential confounders such as hypertension and diabetes, both known to cause glomerular enlargement [41]. The underrepresentation of class III obesity in our cohort may have limited the ability to detect significant differences. The lack of precise information on the duration and severity of diabetes represents a study limitation and may have introduced bias. Nevertheless, in the present study a linear regression model revealed positive correlations between BMI, hypertension, elevated eGFR, and larger glomerular size, supporting the notion of glomerulomegaly as an adaptive response to increased intraglomerular pressure (*P* = .04). This positive association has also been reported in several similar studies, although these cohorts generally included fewer patients (fewer than 100 individuals), patients with more severe obesity, or non-European populations [19, 23, 24, 26]. Despite a lower mean BMI (35 kg/m² vs. 41.7 kg/m²), Praga *et al*. reported a larger mean flocculus diameter (256 ± 24 µm vs. 226 ± 24.6 µm) than Kambham *et al*., suggesting that glomerular size may not correlate directly with obesity severity [21, 20]. In another autopsy series, glomerular enlargement and FSGS were associated with hyperlipidemia but not with BMI, highlighting the limitations of BMI as a marker of renal metabolic stress [42]. Finally, we found no association between glomerulomegaly and OSAS, contrary to Serra *et al*., who reported a higher prevalence of glomerulomegaly in patients with OSAS (51% vs. 28%) among 95 individuals with class III obesity undergoing bariatric surgery [24].

The true prevalence of ORG remains unknown due to its often-subclinical course, the selective use of kidney biopsy in patients with obesity, and the challenge of interpreting proteinuria in the context of hypertension and diabetes. Performing renal biopsy in asymptomatic individuals with obesity raises ethical concerns. However, no serious complications were observed in our cohort compared to patients without or in cohorts with more severe obesity, supporting the safety of renal biopsy in this population and its valuable role in guiding specific therapeutic decisions [23, 25, 26]. Fortunately, in the absence of targeted therapy, misclassification of ORG as hypertensive nephrosclerosis is unlikely to significantly affect CKD management.

This is the first European renal biopsy-based study specifically focused on the spectrum of kidney lesions in patients with obesity. The study strengths include its original design, robust sample size, and the broad geographic referral base of our renal pathology laboratory, which enhances the generalizability of its findings to national clinical practice. Several limitations must be acknowledged. Its retrospective and cross-sectional design included only biopsies performed for clinical indications. Another limitation is that 22% of biopsies were inadequate in patients with obesity, and there were significantly more noncontributive biopsies in this group. As a result, the study may have been underpowered to exclude FSGS lesions; therefore, conclusions regarding the rarity of isolated ORGs should be interpreted with caution. However, this rate of inadequacy was similar in the control group and represents a well-known limitation of renal histological exploration in the context of biopsy. In addition, the use of BMI as a selection criterion introduces selection bias, given its poor correlation with visceral fat and overall metabolic health. Waist circumference, although unable to differentiate visceral from subcutaneous fat, is more strongly associated with insulin resistance and metabolically unhealthy obesity, and may serve as a more appropriate surrogate for adiposity. It may be more relevant in the future to adopt the newer definition, which distinguishes between preclinical obesity without organ dysfunction and clinical obesity with established organ damage [3]. Another limitation concerns the lack of a standardized definition of glomerulomegaly. The threshold of 180 µm, proposed by Kambham based on a small healthy cohort, remains arbitrary [20]. In our own lean controls without diabetes and hypertension (*n* = 37), we identified a slightly lower cut-off of 168 µm. It should be noted that our biopsies were fixed in AFA (a solution of ethyl alcohol, formalin, and acetic acid), which induces greater tissue shrinkage due to the coagulating effect of alcohol. The subjectivity of morphometric assessment may hinder the consistent identification of ORG and its integration into routine pathology practice. Looking forward, the integration of digital pathology tools and artificial intelligence is expected to increase the availability of scanned renal biopsy slides and enable automated, large-scale morphometric analyses. Such approaches will improve the definition of ORG and refine the criteria for glomerulomegaly by expanding the number of biopsies that can be systematically evaluated. In addition, we did not assess ultrastructural changes because electron microscopy was not available, which could have confirmed podocyte injury or other ultrastructural alterations typical of ORG and thereby strengthened the diagnosis. Finally, the lack of follow-up data limited our ability to evaluate renal function trajectories or assess the effect of interventions such as weight loss, RAASi, SGLT2 inhibitors, or GLP1 agonist across obesity phenotypes.

## CONCLUSION

In patients with mild to moderate obesity and kidney disease, ORG is not the predominant cause. The possibility of less common etiologies should prompt nephrologists to consider renal biopsy in this population, given the demonstrated technical feasibility, procedural safety, and diagnostic value. In the absence of a clear alternative diagnosis, ORG should be regarded as a diagnosis of exclusion, warranting greater awareness among clinicians of its recognition and clinical relevance.

## Supplementary Material

sfag092_Supplemental_Files

## Data Availability

The data supporting the findings of this study are available from the corresponding author upon reasonable request.

## References

[1] World Health Organization epidemiological data from 2016.

[2] Fontbonne A, Currie A, Tounian P et al. Prevalence of overweight and obesity in France: the 2020 Obepi-Roche Study by the “Ligue Contre l’Obésité”. JCM. 2023;12:925. 10.3390/jcm12030925. 36769573 PMC9918095

[3] Rubino F, Cummings DE, Eckel RH et al. Definition and diagnostic criteria of clinical obesity. Lancet Diabetes Endocrinol. 2025;13:221–62. 10.1016/S2213-8587(24)00316-4. 39824205 PMC11870235

[4] Garofalo C, Borrelli S, Minutolo R et al. A systematic review and meta-analysis suggests obesity predicts onset of chronic kidney disease in the general population. Kidney Int. 2017;91:1224–35. 10.1016/j.kint.2016.12.013 28187985

[5] Chang AR, Grams ME, Ballew SH et al. Adiposity and risk of decline in glomerular filtration rate: meta-analysis of individual participant data in a global consortium. BMJ. 2019;364:k5301. 10.1136/bmj.k5301 30630856 PMC6481269

[6] Hsu CY, Iribarren C, McCulloch CE et al. Risk factors for end-stage renal disease: 25-year follow-up. Arch Intern Med. 2009;169:342–50. 10.1001/archinternmed.2008.605 19237717 PMC2727643

[7] Stengel B, Tarver-Carr ME, Powe NR et al. Lifestyle factors, obesity and the risk of chronic kidney disease. Epidemiology. 2003;14:479–87. 10.1097/01.EDE.0000071413.55296.c4 12843775

[8] Foster MC, Hwang SJ, Larson MG et al. Overweight, obesity, and the development of stage 3 CKD: the Framingham Heart Study. Am J Kidney Dis. 2008;52:39–48. 10.1053/j.ajkd.2008.03.003 18440684 PMC2531220

[9] Pinto-Sietsma SJ, Navis G, Janssen WMT et al. A central body fat distribution is related to renal function impairment, even in lean subjects. Am J Kidney Dis. 2003;41:733–41. 10.1016/S0272-6386(03)00020-9 12666059

[10] Kramer H, Luke A, Bidani A et al. Obesity and prevalent and incident CKD: the hypertension detection and follow-up program. Am J Kidney Dis. 2005;46:587–94. 10.1053/j.ajkd.2005.06.007 16183412

[11] Ejerblad E, Fored CM, Lindblad P et al. Obesity and risk for chronic renal failure. J Am Soc Nephrol. 2006;17:1695–702. 10.1681/ASN.2005060638 16641153

[12] Vivante A, Golan E, Tzur D et al. Body mass index in 1.2 million adolescents and risk for end-stage renal disease. Arch Intern Med. 2012;172:1644–50. 10.1001/2013.jamainternmed.85 23108588 PMC4941233

[13] Praga M, Hernández E, Herrero JC et al. Influence of obesity on the appearance of proteinuria and renal insufficiency after unilateral nephrectomy. Kidney Int. 2000;58:2111–8. 10.1111/j.1523-1755.2000.00384.x 11044232

[14] Chen HM, Liu ZH, Zeng CH et al. Podocyte lesions in patients with obesity-related glomerulopathy. Am J Kidney Dis. 2006;48:772–9. 10.1053/j.ajkd.2006.07.025 17059996

[15] Bonnet F, Deprele C, Sassolas A et al. Excessive body weight as a new independent risk factor for clinical and pathological progression in primary IgA nephritis. Am J Kidney Dis. 2001;37:720–7. 10.1016/S0272-6386(01)80120-7 11273871

[16] Berthoux F, Mariat C, Maillard N. Overweight/obesity revisited as a predictive risk factor in primary IgA nephropathy. Nephrol Dial Transplant. 2013;28:iv160–6. 10.1093/ndt/gft286 24026246

[17] Othman M, Kawar B, El Nahas AM. Influence of obesity on progression of non-diabetic chronic kidney disease: a retrospective cohort study. Nephron Clin Pract. 2009;113:c16–23. 10.1159/000228071 19590231

[18] Weisinger JR, Kempson RL, Eldridge FL et al. The nephrotic syndrome: a complication of massive obesity. Ann Intern Med. 1974;81:440–7. 10.7326/0003-4819-81-4-440 4416380

[19] Chen HM, Li SJ, Chen HP et al. Obesity-related glomerulopathy in China: a case series of 90 patients. Am J Kidney Dis. 2008;52:58–65. 10.1053/j.ajkd.2008.02.303 18423814

[20] Kambham N, Markowitz GS, Valeri AM et al. Obesity-related glomerulopathy: an emerging epidemic. Kidney Int. 2001;59:1498–509. 10.1046/j.1523-1755.2001.0590041498.x 11260414

[21] Praga M, Hernández E, Morales E et al. Clinical features and long-term outcome of obesity-associated focal segmental glomerulosclerosis. Nephrol Dial Transplant. 2001;16:1790–8. 10.1093/ndt/16.9.1790 11522860

[22] Weibel ER, Gomez DM. A principle for counting tissue structures on random sections. J Appl Physiol. 1962;17:343–8. 10.1152/jappl.1962.17.2.343 14005589

[23] Goumenos DS, Kawar B, El Nahas M et al. Early histological changes in the kidney of people with morbid obesity. Nephrol Dial Transplant. 2009;24:3732–8. 10.1093/ndt/gfp329 19596742

[24] Serra A, Romero R, Lopez D et al. Renal injury in the extremely obese patients with normal renal function. Kidney Int. 2008;73:947–55. 10.1038/sj.ki.5002796 18216780

[25] Salvatore SP, Chevalier JM, Kuo SF et al. Kidney disease in patients with obesity: it is not always obesity-related glomerulopathy alone. Obesity Res Clin Pract. 2017;11:597–606. 10.1016/j.orcp.2017.04.003 28442280

[26] Choung HYG, Bomback AS, Stokes MB et al. The spectrum of kidney biopsy findings in patients with morbid obesity. Kidney Int. 2019;95:647–54. 10.1016/j.kint.2018.11.026 30712921

[27] de Laforcade L, Bobot M, Boffa JJ et al. Kidney biopsy for the diagnosis and treatment of kidney diseases. Recommendations from the French speaking Society of Nephrology (SFNDT) and French National Authority for Health (HAS) 2022. Nephrol Ther. 2024;20:61–80. 10.1684/ndt.2024.68 38379375

[28] Lameire NH, Levin A, Kellum JA et al. Harmonizing acute and chronic kidney disease definition and classification: report of a Kidney Disease: Improving Global Outcomes (KDIGO) Consensus Conference. Kidney Int. 2021;100:516–26. 10.1016/j.kint.2021.06.028 34252450

[29] Loupy A, Haas M, Roufosse C et al. The Banff 2019 Kidney Meeting Report (I): updates on and clarification of criteria for T cell– and antibody-mediated rejection. Am J Transplant. 2020;20:2318–31. 10.1111/ajt.15898 32463180 PMC7496245

[30] WHO European Regional Obesity Report 2022 . Copenhagen: WHO Regional Office for Europe; 2022. Licence: CC BY-NC-SA 3.0 IGO.

[31] Hales CM. Prevalence of obesity and severe obesity among adults: United States, 2017–2018. 2020. 32487284

[32] WHO Expert Consultation . Appropriate body-mass index for Asian populations and its implications for policy and intervention strategies. Lancet. 2004;363:157–63. 10.1016/S0140-6736(03)15268-3 14726171

[33] Tsuboi N, Koike K, Hirano K et al. Clinical features and long-term renal outcomes of Japanese patients with obesity-related glomerulopathy. Clin Exp Nephrol. 2013;17:379–85. 10.1007/s10157-012-0719-y 23135866

[34] Poggio ED, McClelland RL, Blank KN et al. Systematic review and meta-analysis of native kidney biopsy complications.Clin J Am Soc Nephrol. 2020;15:1595–602. 10.2215/CJN.04710420 33060160 PMC7646247

[35] Lemoine S, Guebre-Egziabher F, Sens F et al. Accuracy of GFR estimation in obese patients. Clin J Am Soc Nephro. 2014;9:720–7. 10.2215/CJN.03610413 PMC3974350 24482068

[36] Guebre-Egziabher F, Brunelle C, Thomas J et al. Estimated glomerular filtration rate bias in participants with severe obesity regardless of deindexation. Obesity. 2019;27:2011–7. 10.1002/oby.22574 31579999

[37] López-Martínez M, Luis-Lima S, Morales E et al. The estimation of GFR and the adjustment for BSA in overweight and obesity: a dreadful combination of two errors. Int J Obes. 2020;44:1129–40. 10.1038/s41366-019-0476-z 31641213

[38] VD D'Agati, Chagnac A, de Vries APJ et al. Obesity-related glomerulopathy: clinical and pathologic characteristics and pathogenesis. Nat Rev Nephrol. 2016;12:453–71. 10.1038/nrneph.2016.75 27263398

[39] de Vries APJ, Ruggenenti P, Ruan XZ et al. Fatty kidney: emerging role of ectopic lipid in obesity-related renal disease. Lancet Diabetes Endocrinol. 2014;2:417–26. 10.1016/S2213-8587(14)70065-8 24795255

[40] Wahba IM, Mak RH. Obesity and obesity-initiated metabolic syndrome: mechanistic links to chronic kidney disease. Clin J Am Soc Nephrol. 2007;2:550–62. 10.2215/CJN.04071206 17699463

[41] Hughson MD, Hoy WE, Douglas-Denton RN et al. Towards a definition of glomerulomegaly: clinical-pathological and methodological considerations. Nephrol Dial Transplant. 2011;26:2202–8. 10.1093/ndt/gfq688 21115671 PMC3164445

[42] Verani RR. Obesity-associated focal segmental glomerulosclerosis: pathological features of the lesion and relationship with cardiomegaly and hyperlipidemia. Am J Kidney Dis. 1992;20:629–34. 10.1016/S0272-6386(12)70230-5 1462993

