# Supplementary material for: Kidney biopsy findings in patients with obesity exhibit a wide spectrum of disease entities
Source: Clin Kidney J. 2026 Mar 19;19(4):sfag092. doi: 10.1093/ckj/sfag092 (PMC13129267; doi:10.1093/ckj/sfag092)
Supplement: sfag092_Supplemental_Files [file sfag092_supplemental_files.zip › Supplementary Tables R2-with .docx]

| **Supplementary Table 1: Renal biopsy findings in 103 patients with obesity stratified by obesity class** | | | | | |  |  |
| --- | --- | --- | --- | --- | --- | --- | --- |
|  | **Obesity class** | | | **Overall (N=103)** | **Matched control patients (N=206)** | **p-value^a^** | **p-value^b^** |
|  | **Class I (N= 65)** | **Class II (N=20)** | **Class III (N=18)** |  |  |  |  |
| **ATN** | 22 (34) | 8 (40) | 5 (28) | 35 (34) | 50(24) | 0.7 | 0.07 |
| **ORG (>168 µm)** | 22 (34) | 10 (50) | 8 (44) | 40 (39) | NA | 0.4 | NA |
| **ORG (> 180 µm)** | 14 (22) | 6 (30) | 6 (33) | 26 (25) | NA | 0.5 | NA |
| **Hypertensive nephrosclerosis** | 23 (35) | 7 (35) | 5 (28) | 35 (34) |  | 0.8 | > 0.9 |
| **Diabetic nephropathy** | 9 (14) | 5 (25) | 3 (17) | 17 (17) | 38 (18) | 0.5 | 0.7 |
| **IgA nephropathy** | 11 (17) | 0 (0) | 2 (11) | 13 (13) | 23(11) | 0.14 | 0.7 |
| **Interstitial nephritis** | 7 (11) | 1 (5) | 2 (11) | 10 (9.7) | 19(9) | 0.8 | 0.16 |
| **Lupus nephritis** | 7 (11) | 1 (5) | 2 (11) | 10 (9.7) | 30(15) | 0.8 | 0.3 |
| **TMA** | 5 (7.7) | 0 (0) | 0 (0) | 5 (4.9) | 7(3) | 0.4 | 0.5 |
| **Infection-related GN** | 1 (1.5) | 2 (10) | 1 (5.6) | 4 (3.9) | 1(0.5) | 0.14 | 0.04 |
| **Pauci-immune GN** | 2 (3.1) | 0 (0) | 1 (5.6) | 3 (2.9) | 21(10) | 0.5 | 0.02 |
| **Membranous nephropathy** | 2 (3.1) | 2 (10) | 0 (0) | 4 (3.9) | 18(9) | 0.3 | 0.2 |
| **Primary FSGS** | 2 (3.1) | 1 (5) | 0 (0) | 3 (2.9) | 6(3) | 0.8 | > 0.9 |
| **Minimal Change Disease** | 2 (3.1) | 1 (5) | 0 (0) | 3 (2.9) | 8(4) | 0.8 | 0.8 |
| **MGRS** | 2 (3.1) | 0 (0) | 0 (0) | 2 (1.9) | 7(3) | > 0.9 | 0.7 |
| Values presented are N (%).^a^ p values reflect comparisons between the three obesity groups (class I, class II, and class III), using the Chi-squared test.  **^b^** p values reflect comparisons between patients with obesity and matched controls without obesity usingthe Chi-squared test  ATN: acute tubular necrosis; GN: glomerulonephritis; MGRS: monoclonal gammapathy of renal significance; ORG: obesity related glomerulopathy; Primary FSGS: primary focal segmental glomerulosclerosis; TMA: thrombotic microangiopathy | | | | | |  |  |

| **Supplementary Table 2: Renal biopsy findings in 103 patients with stratified the presence or not of**  Obesity-Related Glomerulopathy **(ORG).** | | |  |
| --- | --- | --- | --- |
| **ORG alone (N=7)** | **ORG with renal disease (n=19)** | **Other lesion alone (N=77)** |  |
| Glomerulomegaly with FSGS (1) | Diabetic nephropathy (n=7) | Diabetic nephropathy (n=10) |  |
| Glomerulomegaly without FSGS (6) | Hypertensive nephrosclerosis (n=10) | Hypertensive nephrosclerosis (n=29) |  |
|  | IgA nephropathy (n=2) | IgA nephropathy (n=11) |  |
|  | GNMP (n=1) | Membranous nephropathy (n=3) |  |
|  | Acute/chronic interstitial nephritis (n=1) | Primary FSGS (n=2) |  |
|  | Lupus nephritis (n=3) | Minimal Change Disease (n=3) |  |
|  | TMA (n=1) | Acute/chronic interstitial nephritis (n=9) |  |
|  |  | GN pauci immune (n=3) |  |
|  |  | Lupus nephritis (n=8) |  |
|  |  | TMA (n=4) |  |
|  |  | Infection related GN (n=4) |  |
|  |  | GNMP (n=3) |  |
|  |  | MGRS (n=2) |  |
| FSGS: Focal Segmental Glomerulosclerosis; GNMP: Glomerulonephritis Membranoproliferative; GN pauci-immune: Pauci-immune Glomerulonephritis; IgA nephropathy: Immunoglobulin A Nephropathy; MGRS: Monoclonal Gammopathy of Renal Significance; ORG: Obesity-Related Glomerulopathy; TMA: Thrombotic Microangiopathy. | | | |
