# Supplementary figures and images for: Kidney biopsy findings in patients with obesity exhibit a wide spectrum of disease entities
Source: Clin Kidney J. 2026 Mar 19;19(4):sfag092. doi: 10.1093/ckj/sfag092 (PMC13129267; doi:10.1093/ckj/sfag092)

## Slide 1
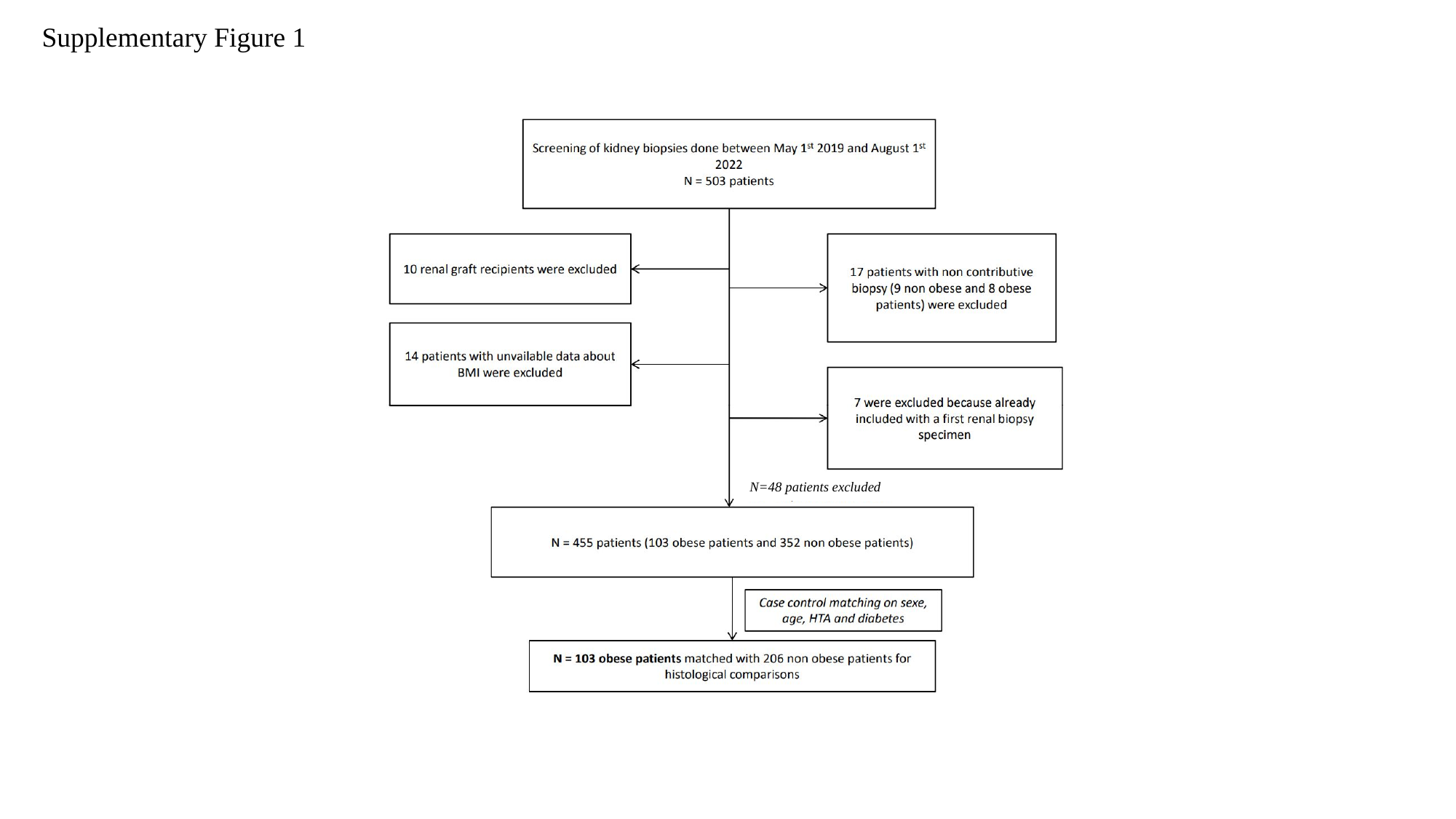

Supplementary Figure 1
N=48 patients excluded

## Slide 2
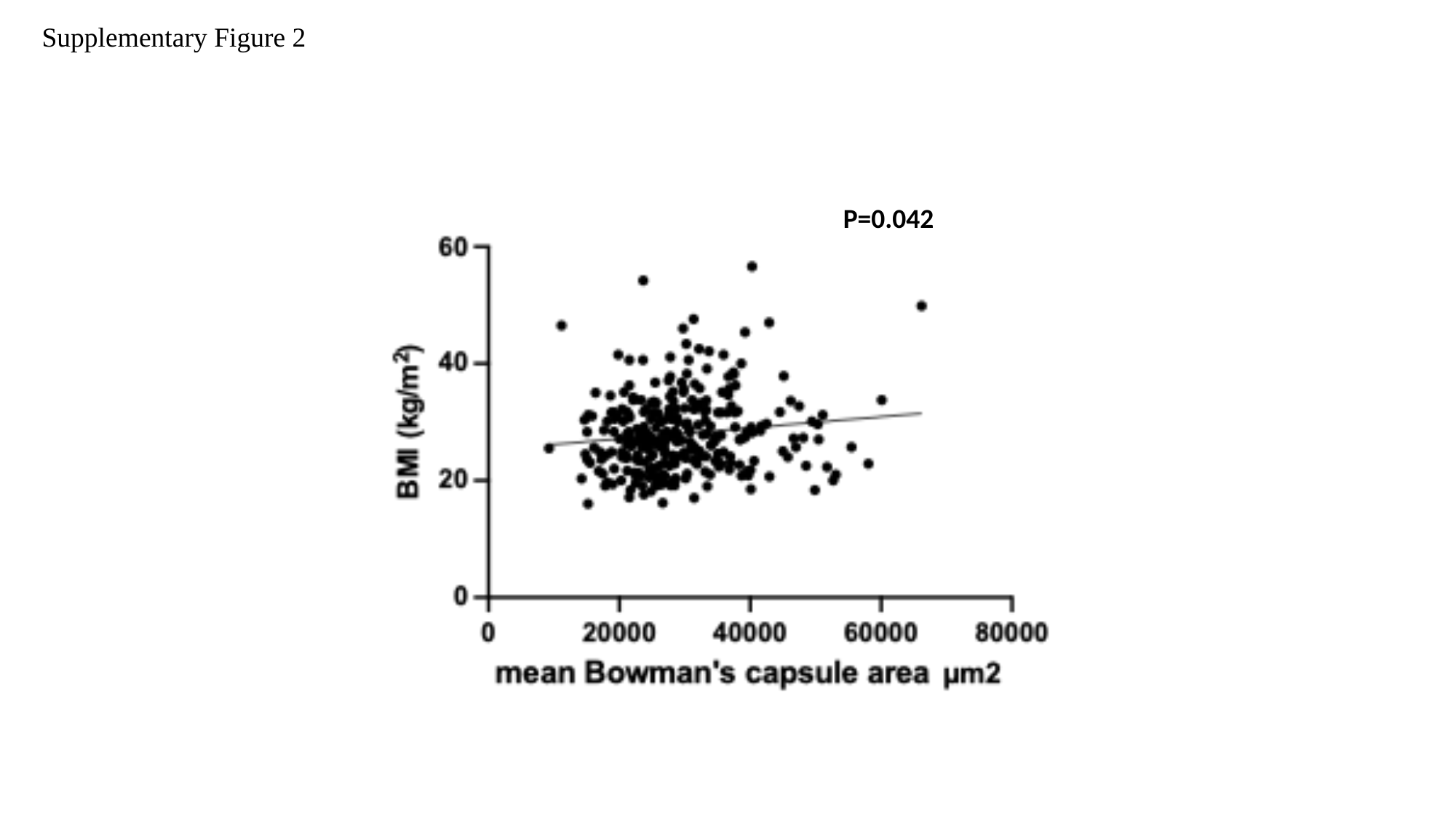

Supplementary Figure 2
P=0.042

Supplement: sfag092_Supplemental_Files [file sfag092_supplemental_files.zip › Supplementary Figure 1.pptx]
